# Perception about Health Applications (Apps) in Smartphones towards Telemedicine during COVID-19: A Cross-Sectional Study

**DOI:** 10.3390/jpm12111920

**Published:** 2022-11-17

**Authors:** Lingala Kalyan Viswanath Reddy, Pallavi Madithati, Bayapa Reddy Narapureddy, Sahithya Ravali Ravula, Sunil Kumar Vaddamanu, Fahad Hussain Alhamoudi, Giuseppe Minervini, Saurabh Chaturvedi

**Affiliations:** 1 Department of Public Health, College of Health Sciences, Saudi Electronic University, Abha 61421, Saudi Arabia; 2 Department of Biochemistry, Apollo Institute of Medical Sciences and Research, Chittoor 517001, India; 3 Department of Public Health, College of Applied Medical Sciences, King Khalid University, Abha 61421, Saudi Arabia; 4 Department of Pharmacy Practice, SRM College of Pharmacy, SRM Institute of Science and Technology, Chennai 600002, India; 5 Department of Dental Technology, College of Applied Medical Sciences, King Khalid University, Abha 61421, Saudi Arabia; 6 Dental Technology Department, College of Applied Medical Science, King Khalid University, Abha 61421, Saudi Arabia; 7 Multidisciplinary Department of Medical-Surgical and Dental Specialties, University of Campania “Luigi Vanvitelli”, Via Luigi de Crecchio 6, 80138 Naples, Italy; 8 Department of Prosthetic Dentistry, College of Dentistry, King Khalid University, Abha 61421, Saudi Arabia

**Keywords:** COVID-19, health applications, telemedicine, perception, consumers, smartphones

## Abstract

Background: The use of health applications (apps) in smartphones increased exponentially during COVID-19. This study was conducted the with the aim to understand the factors that determine the consumer’s perception of health apps in smartphones towards telemedicine during COVID-19 and to test any relation between these factors and consumers towards Telemedicine in India. Methods: This questionnaire-based cross-sectional study was conducted from July 2021 to December 2021 in India. Out of 600 selected participants, 594 responded and in that 535 valid questionnaires were measured. The questionnaire consists of close-ended responses, with the first part consisting of demographic information, the second part consisting of questions associated with consumers’ perceptions and the third part kept for suggestions and complaints. The questionnaire was distributed using digital platforms via WhatsApp or email. A 5-point Likert scale, ranging from strongly agree’ (5) to strongly disagree (1) was used to record responses. Results: Results revealed a high response rate of 90%. The highest score was obtained for the question assessing the satisfaction of the users towards health apps [1175 = 500 (agree-4) + 675 (Strongly agree-5)]. The interface of the app scored very low, showing disagreement (514) with app functionality, and was the most common disadvantage as perceived by patients. The mean scores of reliabilities and vicinity of health services; efficacy and comprehensive health information; development and improvement of health apps and telemedicine (3.24, 3.18, 3.62, 3.49), respectively, show the difference in attraction existing between groups. There is a strong positive correlation between the variables except for efficacy and comprehensive information about health and Telemedicine (−0.249), development and improvement of health apps, and reliability and vicinity of health services (−0.344) which have a negative correlation. Conclusions: The findings of this survey reveal a positive outlook of health apps toward telemedicine. This research also found a strong forecaster of the consumer’s perception of health apps in smartphones towards telemedicine. In the broad spectrum, the future of health app affiliates for telemedicine is better affected by the consumer’s perception of health app efficacy. This study suggests that health app marketers develop more innovative apps to increase usage and help consumers.

## 1. Introduction

Over the past few decades, various new emerging infectious diseases have raised concern about public health and have resulted in different epidemic situations. In recent times, several viral infections are constantly reported worldwide, like the Ebola virus, Nipah virus, coronavirus, influenza virus, Monkeypox virus, etc. [1]. The current published evidence and early reports suggest that the virus that causes COVID-19 is transmitted from person-to-person through close exposure to infected cases through droplet infection like that of many other respiratory diseases. The infection is spread when an infected person is speaking, coughing, singing, or sneezing. These droplets can enter through any natural orifice and mucus membrane in the nearby person’s nose, mouth, or eyes or directly inhale into the lungs [2]. Fomite transmission, although it might not be a predominant mode of transmission, through touching a contaminated surface contributes to the transmission of SARS-CoV-2 to other individuals [3].

People most at risk are healthcare providers [HCP] for acquiring the disease as those who are in contact with places or providing care for patients with COVID-19. Preventing infection among patients and protecting healthcare providers is of paramount importance to any country. As evident from the infection dynamics in almost all countries, a significant patient burden on the health system has, in turn, increased the risk for healthcare staff, especially those dealing with both confirmed and asymptomatic COVID-19 cases directly. Recent outbreaks in hospitals have reinforced that the public and HCP with COVID-19 do not report any symptoms [4]. Unrecognized asymptomatic and pre-symptomatic infections are expected to transmit the disease in various healthcare settings. Based on recent reports from several countries, it was anticipated that the COVID-19 pandemic would follow similar transmission dynamics. It is crucial to protect healthcare providers and their family members from contracting COVID-19 and minimize the interfamilial transmission of COVID-19 [5].

According to the data, as of 3 June 2022, the overall number of COVID-19 cases worldwide is 534,847,110, and the total number of deaths is 6,319,211, and India is one of the top three affected countries with confirmed cases of 43,171,872, and total deaths are 524,651 [6]. Data released by Amnesty International found that globally over 7000 Healthcare professionals [HCP] died due to COVID-19, it is an alarming situation. In the U.S alone, 1077 HCP died, and 573 healthcare providers died in India due to COVID-19 [7]. With the above background, HCPs are a critical group affected by COVID-19. Therefore, telemedicine is one of the best alternatives for healthcare providers to provide quality treatment for patients by protecting the public and themselves [8,9,10]. In COVID-19, HCP self-protection is of utmost importance because it might be a source of cross-infection [11]. Like any conventional treatment modalities of medical support, where any HCP efficiency depends on his/her well-being, in telemedicine success depends intensely on it along with several other factors associated with patients [12,13].

Telemedicine, a term created in the 1970s, is “healing at a distance” [14], implying the practice of information and communication technologies (ICT) to progress patient consequences by growing access to care and medical information [11,15]. Telecommunication technologies can enable healthcare delivery to patients residing in distant areas and facilitate data exchange between healthcare professionals and end-users. Smartphone technology enables mobile computing at the point of care by uniting communication and computation in a handheld-sized device [16]. As per World Bank 2014 data, 68% of people live in rural areas in India [World Bank Data, 2014]. There are nearly 1168.8 million mobile subscribers and 624 million internet users in India as of January 2021 [17,18].

Telemedicine is not a distinct medical field but can be installed by health care providers and specialties [19]. Telemedicine technology can act as remote consultations for patients in rural areas or off-hours [12,20]. The main aim of this study was to understand the factors that determine the consumer’s perception of health applications [apps] in smartphones towards telemedicine and to test any relation between these factors and consumers toward telemedicine. Therefore, it is apparent that the telemedicine concept incorporates the transfer and exchange of information by healthcare professionals using telecommunications [21] for proficient consultation even after even after discharge video link technology, improving patients’ quality of life [22]. There are many apps available for smartphones based on the need, for example, to text your doctor about a chronic condition–Pingmd, for answers to some confounding questions–HealthTap, etc. [Nuq P.A. and Aubert B, 2013] [23].

The Government of India introduced various mobile apps such as the Directory Services App, AIIMS-WHO CC ENBC, HealthyYou Card, HealthyYou EHR, HealthKartPlus, Safe Pregnancy and Birth, Mswasthya, Supervision, and Evidence-Based Care, T.B. Detect, Geo chat, O.B. Insulin, etc. [NHP, 2015] [24]. ZocDoc is a free service mobile app that enables patients to search for doctors, sort by location, specialty, and insurance acceptance, read verified patient reviews and instantly book an appointment online [20]. The Global Med app has an open architecture feature that can work with all effective videoconferencing systems like Zoom, and Google meets for remote clinical consultation, and other telemedicine activities [20]. However, studies that investigate patients’ perceptions towards health apps and challenges, as well as factors associated with poor satisfaction towards telemedicine, via health apps, are limited. Thus, the present study was conducted with the aim to understand the factors that determine the consumer’s perception of health apps in smartphones towards telemedicine during COVID-19 and to test any relation between these factors and consumers towards Telemedicine in India. This study would be of great benefit as the results could be of help in developing guidelines for the future development of health apps, services to be registered in the apps, technological simplicity and complexity of apps depending upon the demographic census and various other factors as studied in this study. The government, information and technology companies could use the results for developing a user-friendly health app.

## 2. Materials and Methods

### 2.1. Study Design and Setting

A cross-sectional study was conducted between July 2021 to December 2021 in India, to understand the factors that determine the consumer’s perception of health apps in smartphones towards telemedicine during COVID-19 and to test any relation between these factors and consumers toward Telemedicine. There has been very little research available, so based on the associated review of the literature, the following hypotheses (H) were tested in this study.

**Hypotheses** **1** **(H1).** *The consumer’s perception of the reliability and vicinity of health services through health apps on smartphones will have a progressive impact on their approach toward telemedicine.*

**Hypotheses** **2** **(H2).** *The consumer’s perception of efficacy and comprehensive information about health from health apps will significantly impact their attitude toward telemedicine.*

**Hypotheses** **3** **(H3).** *The consumer’s perception of the development and improvement of health apps will positively impact their attitude toward Telemedicine.*

### 2.2. Sample Size Estimation

The sample size was calculated as per the formula *n* = z2 pq/e2. Here, “*n*” is the required sample size, “*p*” is incidence (37.3% of patients’ satisfaction towards telemedicine) taken from the study conducted previously [23], q is 1 – *p* (1 – 0.373) = 0.627, z = 1.96 at 95% confidence interval, and e = 5% margin of error. Applying the values to the above formula, the projected least sample size for this study was 360 participants. On the basis of the area to be covered in India we increased the sample size to 600.

### 2.3. Sampling Method 

The Non-probability purposive sampling technique was used to collect the data. In this method, the chief researcher with co-researchers contacted the patients as per their judgment of the use of health apps.

### 2.4. Ethical Concerns

The investigators had assured all the participants about the privacy and confidentiality of their data, and that the personal identification of the participants will not be revealed. The study was performed in accordance with the Declaration of Helsinki and approved by the Institutional Ethics Committee of Apollo Institute of Medical Sciences and Research, Chittoor Andhra Pradesh, India. Ref no IEC08/AIMSR/07/2020 dated 30 May 2020. Consent from the participants was obtained after explaining the purpose and objectives of the study.

### 2.5. Inclusion and Exclusion Criteria 

The present study included patients using at least one health care app on their own mobile and had taken a minimum of one virtual consultation related to COVID problems. Any participant having a health care app but using call centers or other means of consultation like a website or local clinic was excluded from the study.

### 2.6. Data Collection

A cross-sectional quantitative evaluation was done using a structured questionnaire between July 2021 and December 2021 to address the objectives. The questionnaire was cautiously designed and developed to address the purposes of the study. The questionnaire consists of close-ended responses, with the first part consisting of demographic information of the respondents and the second part consisting of questions associated with consumers’ perceptions about health apps towards telemedicine and the third part was kept for suggestions and complaints about the apps. The questionnaire was distributed using digital platforms via WhatsApp or email.

All measures were evaluated on a Likert scale, ranging from strongly agree’ [5] to strongly disagree [1]. At the end of the survey, the total score was considered. A higher score in the patient response to the questionnaire indicated higher satisfaction with telemedicine services.

A total of 600 participants were selected out of which six refused thus 594 respondents participated. However, after the data collection process, 535 [90%] valid questionnaires were measured in the final analysis, and the response rate was 90%.

The responses were carefully captured and coded in SPSS 21.0 statistical package software for analysis. A pilot survey was conducted among 26 respondents to check the reliability of the measures. Cronbach’s alpha was used for measuring the internal consistency, i.e., the validity and reliability of the data, and found Cronbach’s alpha is 0.84, which is suitable for gathering data for primary research.

## 3. Results

**Figure 1:**
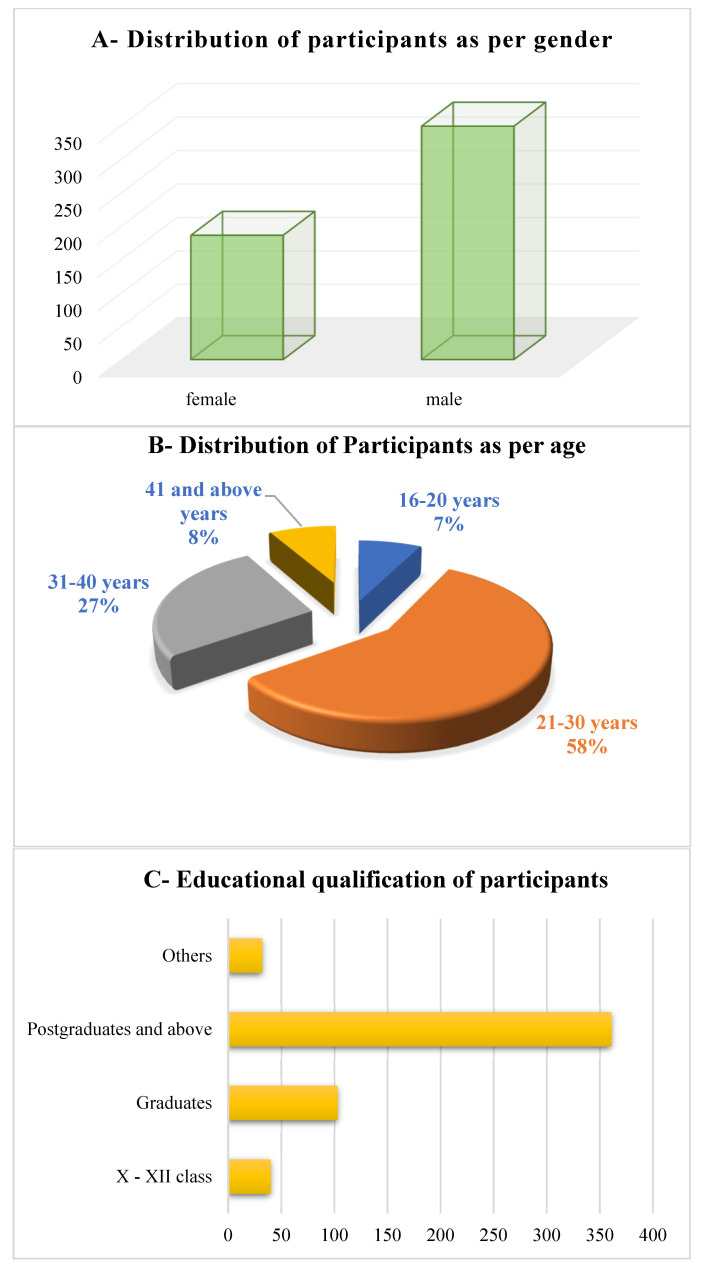

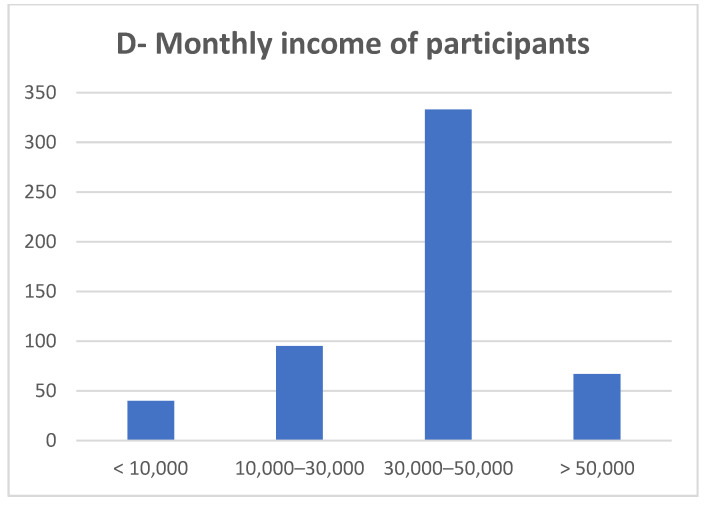
(**A**–**D**) Demographic information of respondents.

The social demographic features of the respondents presented in Figure 1 consist of males [65.2%] and females [34.8%] from a sample size of 535. The highest number of respondents are between the age group 21 and 30 years [57.8%], followed by 31–40 years [26.7%], above 41 years [8.1%], and 16–20 years [7.4%]. The maximum number of respondents have an educational qualification of post-graduation [67.4%], followed by graduates [19.3%]; X and XII classes [7.4%] and [5.9%] others who are having basic education below the ninth class. The income level of respondents is also analyzed which constitutes [62.2%] of them earning between 30,000 and 50,000 followed by [17.8%] of respondents earning between 10,000 to 30,000; above 50,000 by [12.6%] and [7.4%] below 10,000 INR.

Results revealed a high response rate of 90%. The highest score was obtained for the question assessing the satisfaction of the users towards health apps [1175 = 500 (agree-4) + 675(Strongly agree-5)]. The interface of the app scored very low showing disagreement (514) with the apps functionality and was the most common disadvantage as perceived by patients. The correlation coefficients among the variables such as reliability and vicinity of health services; efficacy and comprehensive health information; development and improvement of health apps and telemedicine are shown in Table 1. The mean scores of reliabilities and Vicinity of health services; efficacy and comprehensive health information; development and improvement of health apps and Telemedicine (3.24, 3.18, 3.62, 3.49), respectively, show the difference in attraction existing between groups. There is a strong positive correlation between the variables except for efficacy and comprehensive information about health and Telemedicine (−0.249), development and improvement of health apps, and reliability and vicinity of health services (−0.344) which have a negative correlation. Still, there is a statistically significant relationship among all variables, which is lower than the level of significance [α = 0.01] except between efficacy and comprehensive information about health and telemedicine. Reliability and vicinity of health services through health apps on smartphones were the most vital related factor to telemedicine.

### Multiple Regressions

As shown in Table 2, the regression analysis helps to predict the three dependent variables for telemedicine. In this, the value of the coefficient of determination [R square value] is 0.289, i.e., there is a 28.9% variation in Telemedicine. The standardized coefficient [beta] for the independent variables Reliability and Vicinity of health services; Efficacy and Comprehensive health information; Development and Improvement of health apps is [0.524, −0.136, 0.789], respectively. There is a significant relation between Reliability and Vicinity of health services [α = 0.00], Efficacy and Comprehensive health information [α = 0.039], Development and Improvement of health apps [α = 0.025] with Telemedicine which is lower than the level of statistical significance [α = 0.05]. 

## 4. Discussion

Recent advances in telecommunications such as smartphones and wireless patient monitoring devices improved the delivery of healthcare services [16]. In the coming years, it is evident that mobile health apps will play a significant role in revolutionizing the healthcare industry [25]. The corona pandemic has changed the way of life, and because of its contagious nature through respiratory droplets, even the Health Care Practitioners [HCP] are no exception to this infection. Numerous studies framing various approaches have been presented for infection prevention and control that help in reducing the exposure risk, for example, face mask-wearing, covering of the mouth and nose, regular hand washing, use of hand sanitizer containing at least 60% alcohol, maintaining social distancing and refraining from the contact of unwashed hands with eyes, nose, and mouth [26]. Disease monitoring, surveillance of infections, and disease reporting are the key to protecting healthcare workers during the COVID-19 pandemic. Meanwhile, reducing the face-to-face service for healthcare apps present on mobile plays an important role and it helps not only patients but also healthcare workers can contact patients through telecommunication tools for triaging, assessing, and caring for all patients [27]. Telehealth with various features like the use of live video conferencing or a simple mobile call allows healthcare professionals to question and gather essential information, triage patient and supply consultation, or if a person can continue to self-monitor symptoms at home while recovering. This video conferencing can also be pragmatic for monitoring regular health check-ups such as blood sugar levels, blood pressure and oxygen level rate needed in a home. Therefore, health apps on mobile phones come as a rescue for the patients and HCP to provide treatment, advice, suggestions, consultations, etc., by avoiding close contact with vulnerable individuals in healthcare settings.

Indian government officials launched a remote consultation network that can be carried out via internet or telephone consultations in a safe setting to safeguard the constant provision of health care services and to reduce the hazard of cross infections. Additionally, the National Health Commission of India has published several online guidelines and free electronic books about COVID-19 with the aim of helping the progress of people’s emergency interventions, and safety, and improving the quality and effectiveness of emergency interventions [28,29]. In a previous study, it had been recommended that telehealth has numerous benefits in providing even allergy and immunology services such as limiting the contact of health professionals with potentially infected patients and access to rapid evaluation for COVID-19 infection [30]. In addition to taking actions to guard the health and safety of patients, staff should take mobile health technology to develop staffing plans and carry out billing for healthcare services.

The present study results showed that consumers’ attitude toward telemedicine is affected by the variables discussed in this paper. The findings suggest a strong positive correlation between the variables, except for efficacy; comprehensive information about health from health apps will significantly impact attitudes towards Telemedicine. Reliability and vicinity of health services through health apps on smartphones were the most vital related factor to telemedicine. Personalized healthcare, patient-centric care, and enhanced communication between patients and healthcare professionals are the key aspects that can be supported and enabled using health apps. Questions such as whether the app improved my access to healthcare services, the easy interface of the app, the app would be useful for my health and well-being, Overall, I am satisfied with health apps provided information about consumers satisfaction. Various devices such as smartphones, tablets, and computers use mobile communication services for patient monitoring and delivery of services [30,31].

The demographic factors studied in the present study had a noteworthy association with the consumers’ assessments of health apps. The consumers’ perceptions varied based on demographic factors like age, education, income, etc. [32,33]. like older users had marked more for useful ness of these apps, which may be due to less mobility during old age, so these apps obtain the required information easily. These findings are in correlation with the study conducted in the United Arab Emirates which reveals that the higher the age, the more the users perceive the apps to be advantageous [34]. This reveals that health app makers need to consider the Indian population group while designing the apps in relation to the factors studied in the study.

As per a previous study in the USA, calls and electronic health records (EHR) can facilitate patient exposure time and treatment even without face-to-face visits and also help in decision-making process among healthcare workers [35]. A similar study conducted by Alhajri et al. in 2021 at Abhudhabi reported the perception of physicians towards telemedicine. It was reported that physicians were preferring the health apps and were willing to continue them, as the amount of time required in documentation is less which allowed them to consult more patients also the recall, rearrangement and scheduling of medicines become easy. Conversely, an improper diagnosis or misdiagnosis because of only virtual symptoms-based examination were the drawbacks noticed by the physicians. Additionally, healthcare professionals were preferring health apps allowing both audio-video modes of examination and communication compared to audio care alone [36].

Generally, the impact of telehealth during the COVID-19 pandemic in averting morbidity and avoiding of presence the public from high-risk areas such as hospital premises was significant. Additionally, elderly people can access health services by using electronic devices. These days, a suitable adaptation of local systems with changes regarding payment and coordination of services are major barriers to the large-scale use of telehealth to deal with COVID-19 infection. Eventually, progress can be made in preventing and controlling the COVID-19 pandemic through the further training of healthcare workers and the public on how to make the most of telehealth tools, revisiting traditional definitions of clinical practice, and using closed online platforms. Another study that determined telemedicine’s benefits and drawbacks among physicians in Saudi Arabia reported that 1/3rd of the participants affirmed that telemedicine improved the efficiency of therapeutic intervention and 44% alleged that virtual care enhanced patient care. However, the physicians described virtual care as not the replacement for physical care and consultation and it can be used mainly for stable patients from remote areas [37].

This research has enormous implications for smartphones and internet users as the world is shifting from a conventional marketplace toward e-commerce significantly faster. India is also emerging as a hub for e-marketers and online health consultancies because of its vast internet population of about 80 million [24]. This study will give all health app marketers an overview of the factors that impact the consumer’s attitude. The advancement in technology became affordable and convenient for health care to reach out to remote communities [38,39].

### 4.1. Related Work

During COVID-19, an enormous amount of work had been done to provide better care and support to the needy. In the field of telemedicine and artificial intelligence, various authors conducted various studies and research. Tiwari et al. [38] published a paper to see how Machine Learning (ML) algorithms and applications are used in the COVID-19 inquiry and for other purposes. They reported that ML has advocated a wide range of intelligence-based approaches, frameworks, and equipment to cope with the issues of the medical industry. Khemasuwan and Colt Applications and challenges of AI-based algorithms in the COVID-19 pandemic [39]. Sorkhabi, et al. described a systematic approach for pre-processing electronic health records for mining and mentioned that to extract more precise and reliable knowledge we must pre-process EHRs [40]. This approach could be of great use in telemedicine services. Piri et al. [41] described and demonstrated the use of the artificial gorilla troop optimization (DAGTO) techniques and performed a case study with COVID-19 samples to extract the critical factors related to it. 

### 4.2. Limitations

The limitations of the present study included the 535 sample size, as it cannot be considered an absolute representative of the whole population of India. Even though the research occurred in India, there is a broader research scope for a multinational sample study, and as 57.77% of the respondents were between 21 and 30 years old, the results cannot be generalized. This study used a non-probability purposive sampling method to recruit the participants, so the limitations associated with this method of sampling are applicable to the present study. Consumers’ attitudes may vary based on personal and environmental factors that hinder generalizing the research findings [30]. Managerial implications of this research can differ from using health apps as a new main generator for their commerce and empowering managers and marketers to project health apps that can influence consumers [42,43,44,45].

## 5. Conclusions and Future Work

From the results of the study, it can be concluded that outpatient telemedicine clinics provided better and easy treatment opportunities to patients as more than half of the respondents were satisfied with these clinics. Young, highly educated and economically sound respondents accept and prefer to use these apps more. The technical expertise required with the use of apps was reported as a major disadvantage, even though the respondents desired to use these health apps and telemedicine clinics even after the COVID-19 pandemic. This clearly depicts that technological implementation and advancements in the form of apps have created enthusiasm and awareness among the Indian population and transformed them towards digital care. 

For the future, it could be recommended that health app development should be designed and monitored by a health professional and patient-representing team so that more user-friendly apps can be produced with more focus on the technical issues reported by patients and their caretakers. Future research is recommended on both patient and provider experience and satisfaction with telehealth and associated health apps, to see whether their perspective toward telehealth has changed over time as they become more comfortable and familiar with the system. Additionally, demographic variables used in this study should be considered in future public awareness campaigns for the use of health apps. This becomes mandatory to conduct and evaluate the continuously adding health apps in the market and providing various services. The government and app-developing companies should try to integrate telehealth apps with other electronic health platforms so that it becomes easy for patients and HCP to maintain complete health records. A centralized code system for apps for each user so the data can be assessed by HCP anytime anywhere for better accuracy of medical assessments and quality of care. Regular monitoring and auditing of apps by authorities is recommended for quality improvement purposes. It should be made mandatory that the devices and networks are secure and safe to protect data privacy and confidentiality. Modifications of apps post-COVID-19 and finally the demonstration/tutorial video for patients and HCP to use the apps effectively.

## Figures and Tables

**Table 1:** Correlation Coefficients between Variables.

	Mean	S.D ^#^		R&V	E&C	D&I	TM
R&V	3.24	0.742	Pearson Correlation	1	0.422	−0.344	0.820
			Sig. [2-tailed]		0.001 *	0.001 *	0.000 *
			N	135	135	135	135
E&C	3.18	0.718	Pearson Correlation	0.422	1	0.415	−0.249
			Sig. [2-tailed]	0.001		0.002 *	0.004 *
			N	135	135	135	135
D&I	3.62	0.836	Pearson Correlation	−0.344	0.415	1	0.706
			Sig. [2-tailed]	0.001 *	0.002 *		0.000 *
			N	135	135	135	135
TM	3.49	0.828	Pearson Correlation	0.820	−0.249	0.706	1
			Sig. [2-tailed]	0.000 *	0.0048	0.000 *	
			N	135	135	135	135

R&V = Reliability and Vicinity of health services. E&C = Efficacy and Comprehensive information about health. D&I = Development and Improvement of health apps. TM = Telemedicine. * Correlation is significant at the 0.01 level [2-tailed]. ^#^ S.D = Standard deviation.

**Table 2:** Regression analysis.

Independent Variables	Beta	t	Sig.	R	R Square	Adjusted R Square
Constant]		2.542	0.005	0.553 ^a^	0.289	0.279
Reliability and Vicinity of health services	0.524	6.687	0.000			
Efficacy and Comprehensive information about health	−0.136	−2.118	0.039			
Development and Improvement of health apps	0.789	2.867	0.025			

^a^ Predictors: [Constant], R&V, E&C, D&I.

## Data Availability

Data sharing is not applicable to this article.
